# Evaluation of Optimal Threshold of Neutrophil-Lymphocyte Ratio and Its Association With Survival Outcomes Among Patients With Head and Neck Cancer

**DOI:** 10.1001/jamanetworkopen.2022.7567

**Published:** 2022-04-15

**Authors:** Sung Jun Ma, Han Yu, Michael Khan, Jasmin Gill, Sharon Santhosh, Udit Chatterjee, Austin Iovoli, Mark Farrugia, Hemn Mohammadpour, Kimberly Wooten, Vishal Gupta, Ryan McSpadden, Moni A. Kuriakose, Michael R. Markiewicz, Wesley L. Hicks, Mary E. Platek, Mukund Seshadri, Andrew D. Ray, Elizabeth Repasky, Anurag K. Singh

**Affiliations:** 1 Department of Radiation Medicine, Roswell Park Comprehensive Cancer Center, Buffalo, New York; 2 Department of Biostatistics and Bioinformatics, Roswell Park Comprehensive Cancer Center, Buffalo, New York; 3 Jacobs School of Medicine and Biomedical Sciences, University at Buffalo, The State University of New York, Buffalo; 4 University at Buffalo, The State University of New York, Buffalo; 5 Department of Immunology, Roswell Park Comprehensive Cancer Center, Buffalo, New York; 6 Department of Head and Neck Surgery, Roswell Park Comprehensive Cancer Center, Buffalo, New York; 7 Department of Oral and Maxillofacial Surgery, School of Dental Medicine, University at Buffalo, The State University of New York, Buffalo; 8 Department of Neurosurgery, Department of Surgery, Jacobs School of Medicine and Biomedical Sciences, University at Buffalo, The State University of New York, Buffalo; 9 Department of Nutrition and Dietetics, D’Youville College, Buffalo, New York; 10 Department of Oral Oncology, Roswell Park Comprehensive Cancer Center, Buffalo, New York; 11 Department of Cancer Prevention and Control, Roswell Park Comprehensive Cancer Center, Buffalo, New York

## Abstract

**Importance:**

Given the role of inflammation in cancer progression, neutrophil-lymphocyte ratio (NLR) from peripheral blood has been suggested as a readout of systemic inflammation and a prognostic marker in several solid malignant neoplasms. However, optimal threshold for NLR in US patients with head and neck cancer remains unclear.

**Objective:**

To evaluate the optimal NLR threshold as a potential prognostic biomarker for survival outcomes.

**Design, Setting, and Participants:**

This retrospective cohort study was conducted at a single institution. Participants included 496 patients with nonmetastatic head and neck cancer who underwent chemoradiation from April 2007 to March 2021. Statistical analysis was performed from September to December 2021.

**Exposures:**

High vs low NLR.

**Main Outcomes and Measures:**

Overall survival (OS) and cancer-specific survival (CSS).

**Results:**

A total of 496 patients (411 male patients [82.9%]; 432 White patients [87.1%]; 64 patients with other race or ethnicity [12.9%]; median [IQR] age, 61 [55-67] years) were identified. Median (IQR) follow-up was 44.4 (22.8-74.0) months. Thresholds of NLR for both OS and CSS were 5.71. High NLR above 5.71 was associated with worse OS (adjusted hazard ratio [aHR], 1.97; 95% CI, 1.26-3.09; *P* = .003) and CSS (aHR, 2.33; 95% CI, 1.38-3.95; *P* = .002). On logistic multivariable analysis, patients were more likely to have high NLR if they had higher T and N staging (T3-4: aOR, 4.07; 95% CI, 1.92-9.16; *P* < .001; N2: aOR, 2.97; 95% CI, 1.04-9.17; *P* = .049; N3: aOR, 11.21; 95% CI, 2.84-46.97; *P* < .001), but less likely if they had a good performance status (Karnofsky Performance Status 90-100: aOR, 0.29; 95% CI, 0.14-0.59; *P* < .001). Among 331 patients (66.7%) with available human papillomavirus (HPV) data, high NLR was not associated with OS (HPV-negative: aHR, 2.46; 95% CI, 0.96-6.31; *P* = .06; HPV-positive: aHR, 1.17; 95% CI, 0.38-3.56; *P* = .78) and CSS (HPV-negative: aHR, 2.55; 95% CI, 0.81-7.99; *P* = .11; HPV-positive: aHR, 1.45; 95% CI, 0.44-4.76; *P* = .54).

**Conclusions and Relevance:**

High NLR was associated with worse survival. Patients with substantial disease burden and poor performance status were more likely to have high NLR. These findings suggest that further studies would be warranted to investigate the role of such prognostic marker to identify patients at risk to tailor interventions.

## Key Points

**Question:**

What is an optimal threshold of neutrophil-lymphocyte ratio (NLR) as a biomarker for survival outcomes in patients with head and neck cancer who underwent chemoradiation?

**Findings:**

In this cohort study involving 496 patients, the NLR threshold was 5.71 based on maximizing log-rank test statistic. With statistical significance, high NLR above 5.71 was associated with worse overall and cancer-specific survival, and poor performance status and higher disease burden were associated with high NLR.

**Meaning:**

These findings suggest that high neutrophil-lymphocyte ratio could be an independent, adverse prognostic factor, and further studies would be warranted to tailor treatments among high-risk patients.

## Teaser

This cohort study investigates the optimal neutrophil-lymphocyte ratio (NLR) threshold as a potential prognostic biomarker for survival outcomes among US patients with head and neck cancer.

## Introduction

Inflammation plays a major role in cancer progression.^1^ Emerging biomarkers of systematic inflammation, such as elevated neutrophil-lymphocyte ratio (NLR), have been shown to be prognostic in many solid tumors.^2^ Tumor cells were shown to release cytokines to stimulate the bone marrow to increase the number of neutrophils,^3,4,5^ which in turn release cytokines promoting angiogenesis and metastasis.^6,7,8,9,10,11^ Among patients with head and neck cancers, elevated NLR is an adverse prognostic marker for survival outcomes in multiple meta-analyses.^12,13,14,15,16,17^

However, studies included in such meta-analyses were heterogeneous in patient demographics and treatment characteristics suggesting the mixed strength of association between NLR and survival outcomes.^17^ For example, NLR has been shown to change after induction chemotherapy and head and neck surgery,^18,19^ and NLR values may differ based on racial and ethnic backgrounds.^20^ In addition, the clinical utility of NLR may be challenging, because its optimal threshold remains unclear based on prior studies using its median values or predefined thresholds to stratify high vs low NLR.^17^ Furthermore, the majority of these studies were performed outside the United States.^17^ Given geographic heterogeneity in the prevalence of human papillomavirus (HPV)^21^ and that of other risk factors including smoking and alcohol intake,^22,23^ NLR values may vary based on such lifestyle factors^24^ and findings from such studies may not be generalizable to patients in the United States.^20,24^ To address this knowledge gap and inform clinicians to identify such potentially high-risk patients, we performed a single-institution, observational cohort study of patients treated with chemoradiation in the United States to evaluate the association of NLR and survival outcomes.

## Methods

This cohort study was approved by the Roswell Park Comprehensive Cancer Center institutional review board, and informed consent was waived because the research met the criteria for minimal risk to the study participants. We followed the Strengthening the Reporting of Observational Studies in Epidemiology (STROBE) reporting guideline.

Our retrospective database was built including all patients with primary head and neck cancer who underwent radiation therapy at the Roswell Park Comprehensive Cancer Center between January 2005 and April 2021. Patients were included for analysis if they were diagnosed with nonmetastatic head and neck cancer treated with curative-intent definitive chemoradiation receiving 70 Gy to gross disease and 56 Gy to elective neck lymph nodes. Intensity modulated radiation therapy (IMRT) was performed for all patients in this cohort as previously described.^25^ NLR was obtained from routine complete blood counts (CBC) with differentiation, and patients with unknown NLR were excluded.

In addition to NLR prior to radiation therapy, other variables of interest included age, self-reported gender, self-reported race, smoking history, Karnofsky Performance Status (KPS), number of comorbidities, primary cancer site, cancer staging based on the American Joint Committee on Cancer (AJCC) 7th edition, HPV status, and chemotherapy agent. These variables were included for all of our multivariable analysis (MVA) models. All missing values were coded as unknown for analysis. Among patients who self-reported other racial and ethnic backgrounds, they included African American, American Indian or Alaska Native, Asian, Hispanic, and those who were unknown or declined to answer. These racial and ethnic categories were combined as a single group prior to performing our analyses, because it would be difficult to show meaningful differences in outcomes owing to small subgroup sample sizes. The primary end point of this study was overall survival (OS) and cancer-specific survival (CSS), defined as the time intervals from diagnosis to any death or last follow up and head and neck cancer-related death or last follow up, respectively.

### Statistical Analysis

A threshold for NLR was determined using an outcome-based method by maximizing the log-rank test statistic and the survival differences,^26^ as previously shown in other disease sites.^27,28,29^ Such threshold was evaluated for both OS and CSS separately, and patients were then stratified into 2 cohorts by above vs below the threshold for their NLR values. Fisher exact test and Mann-Whitney *U* test were performed to compare baseline characteristics as appropriate. Kaplan-Meier method and log-rank tests were performed to evaluate survival outcomes. Cox MVA was used to identify variables associated with survival outcomes. Logistic MVA was performed to identify factors associated with high NLR. A subgroup analysis with Cox MVA was also performed among patients with available HPV data.

All statistical tests were 2-sided and *P* values lower than .05 were considered statistically significant. Statistical analyses were performed using R version 4.1.2 (R Project for Statistical Computing) from September to December 2021.

## Results

**Figure 1.**
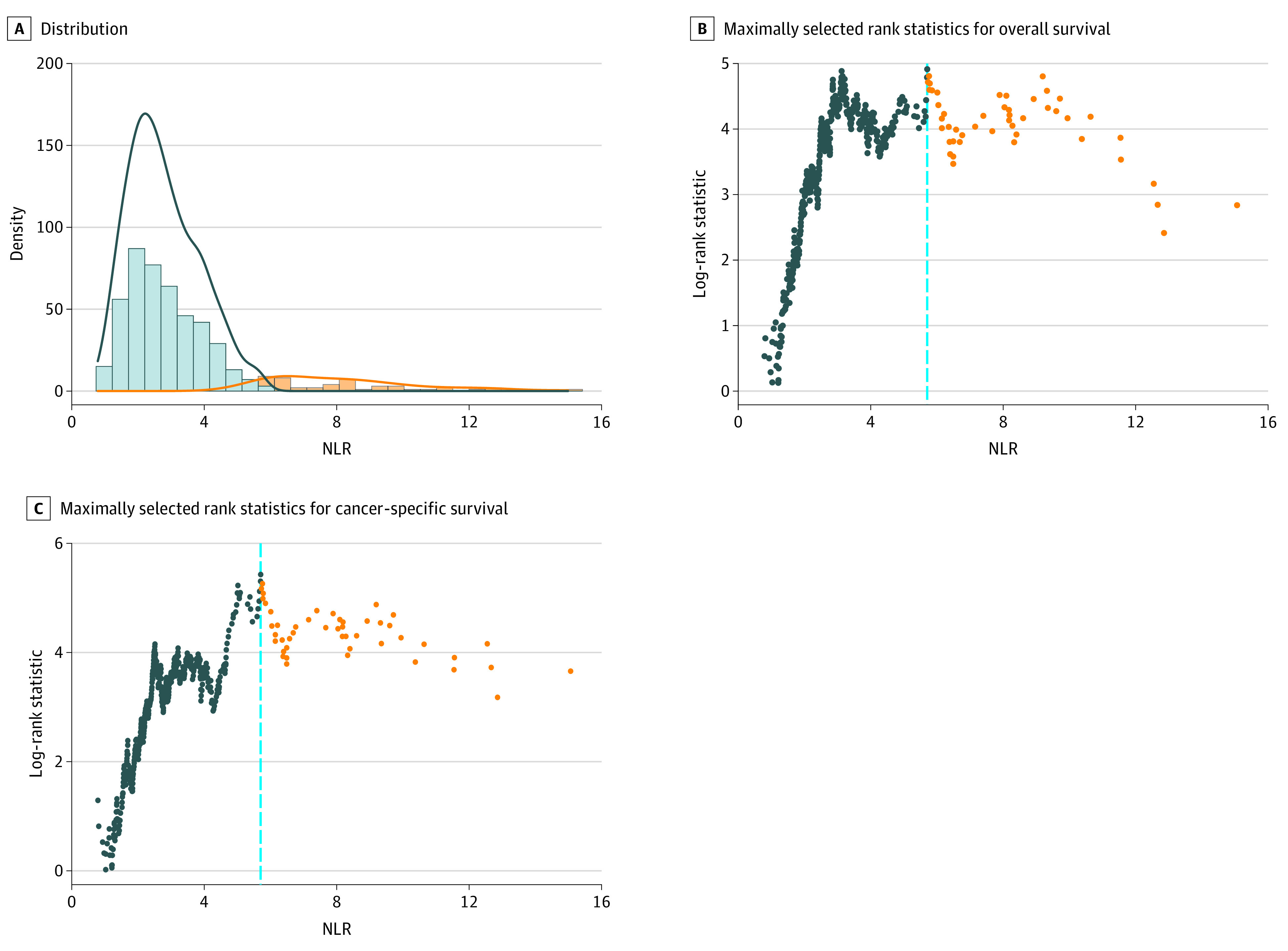
Distribution of Neutrophil-Lymphocyte Ratio (NLR) and Threshold Evaluation Using Maximum Log-Rank Test Statistic

Among the total of 496 patients who met the criteria for the study, 411 (82.9%) identified as male; 432 (87.1%) identified as White, 64 (12.9%) identified as other race or ethnicity; and the median (IQR) age was 61 (55-67) years (Table 1). The majority of patients were diagnosed with oropharyngeal cancer (n = 276; 55.6%) and underwent definitive chemoradiation with cisplatin (n = 419; 84.5%) between April 2007 and March 2021. Median (IQR) follow-up was 44.4 (22.8-74.0) months. Median (IQR) NLR was 2.8 (2.1-3.9) (Figure 1). Four of 496 patients (0.8%) had missing values on KPS, and 165 out of 496 patients (33.3%) had missing values on HPV status in part owing to either having nonoropharyngeal cancer or being diagnosed prior to the routine use of HPV testing (Table 1).^30^ Of all patients, 71 patients (7.9%) were lost to follow up.

**Table 1.** Baseline Characteristics

Characteristic	Patients, No. (%)			*P* value
	All (N = 496)	Low NLR (N = 444)	High NLR (N = 52)	
Gender				
Male	411 (82.9)	373 (84.0)	38 (73.1)	.05
Female	85 (17.1)	71 (76.0)	14 (26.9)	
Smoker				
Never	125 (25.2)	117 (26.4)	8 (15.4)	.13
Current	96 (19.4)	82 (18.5)	14 (26.9)	
Former	275 (55.4)	245 (55.2)	30 (57.7)	
Age, y				
<65	344 (69.4)	305 (68.7)	39 (75.0)	.43
≥65	152 (30.6)	139 (31.3)	13 (25.0)	
Year of radiation				
2014 or earlier	259 (52.2)	230 (51.8)	29 (55.8)	.66
2015 or later	237 (47.8)	214 (48.2)	23 (44.2)	
KPS				
<90	135 (27.2)	107 (24.1)	28 (53.8)	<.001
90-100	357 (72.0)	334 (75.2)	23 (44.2)	
Not available	4 (0.8)	3 (0.7)	1 (1.9)	
Race				
White	432 (87.1)	387 (87.2)	45 (86.5)	.83
Other^a^	64 (12.9)	57 (12.8)	7 (13.5)	
Comorbidity				
0	77 (15.5)	68 (15.3)	9 (17.3)	.01
1	109 (22.0)	97 (21.8)	12 (23.1)	
2	81 (16.3)	65 (14.6)	16 (30.8)	
3	107 (21.6)	103 (23.2)	4 (7.7)	
>3	122 (24.6)	111 (25.0)	11 (21.2)	
Site				
Oropharynx	276 (55.6)	239 (53.8)	27 (51.9)	.51
Larynx	115 (23.2)	100 (22.5)	15 (28.8)	
Oral cavity	12 (2.4)	10 (2.3)	2 (3.8)	
Other	93 (18.8)	85 (19.1)	8 (15.4)	
T staging				
1-2	255 (51.4)	241 (54.3)	14 (26.9)	<.001
3-4	241 (48.6)	203 (45.7)	38 (73.1)	
N staging				
0	97 (19.6)	88 (19.8)	9 (17.3)	.10
1	52 (10.5)	46 (10.4)	6 (11.5)	
2	307 (61.9)	279 (62.8)	28 (53.8)	
3	40 (8.1)	31 (7.0)	9 (17.3)	
HPV				
Negative	92 (18.5)	77 (17.3)	15 (28.8)	.04
Positive	239 (48.2)	222 (50.0)	17 (32.7)	
Not available	165 (33.3)	145 (32.7)	20 (38.5)	
Chemotherapy				
Cisplatin	419 (84.5)	381 (85.8)	38 (73.1)	.02
Other	77 (15.5)	63 (14.2)	14 (26.9)	

Abbreviations: HPV, human papillomavirus; KPS, Karnofsky performance status; NLR, neutrophil-lymphocyte ratio.

^a^ The other category for race and ethnicity included African American, American Indian or Alaska Native, Asian, Hispanic, and those who were unknown or declined to answer.

**Figure 2.**
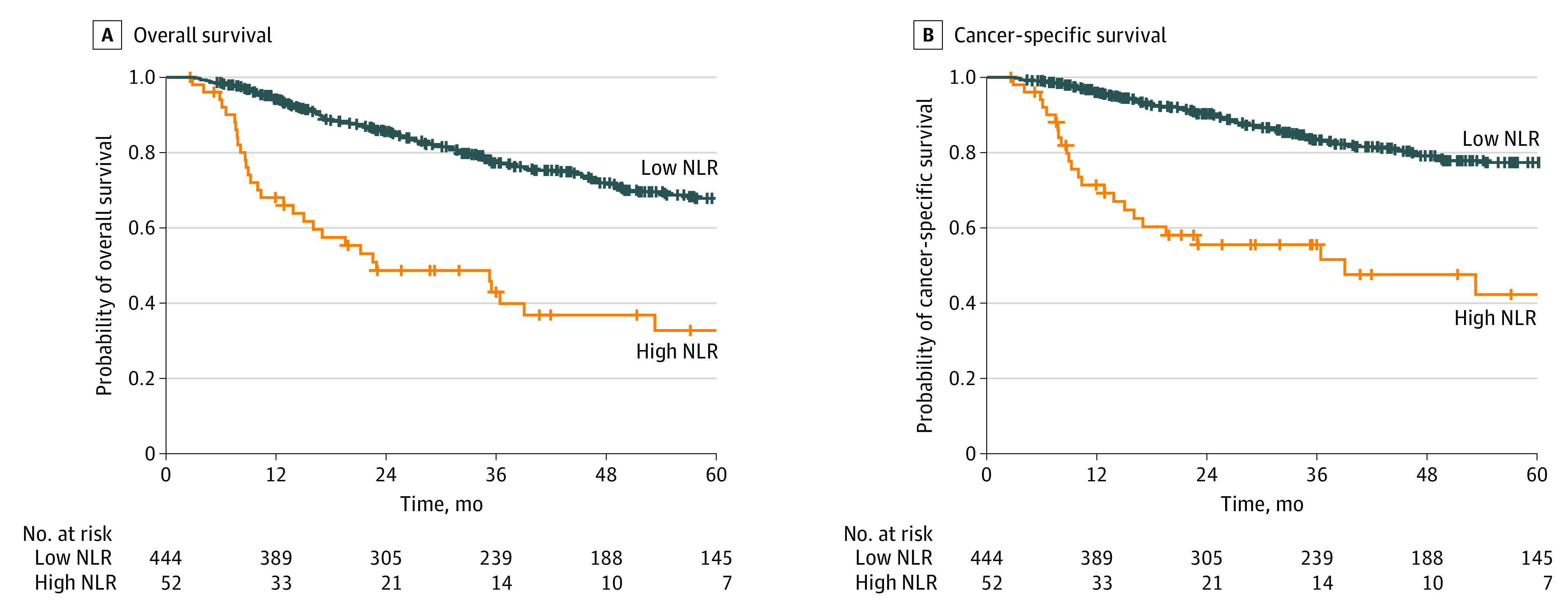
Kaplan-Meier Curve for Overall and Cancer-Specific Survival Outcomes for High vs Low Neutrophil-Lymphocyte Ratio (NLR)

Thresholds of NLR for both OS and CSS were determined to be 5.71 (Figure 1). OS and CSS at 3 years were 77.3% (95% CI, 73.1%-81.7%) and 83.4% (95% CI, 79.5%-87.4%) for the low NLR cohort (*P* < .001); they were 43.0% (95% CI, 30.6%-60.3%) and 55.6% (95% CI, 42.9%-71.9%) for the high NLR cohort (*P* < .001) (Figure 2). On Cox MVA, high NLR was associated with worse OS (adjusted hazard ratio [aHR], 1.97; 95% CI, 1.26-3.09; *P* = .003) and CSS (aHR, 2.33; 95% CI, 1.38-3.95; *P* = .002). In addition, current smoking status, older age, poor KPS, and higher T and N staging were associated with survival outcomes (Table 2).

**Table 2.** Cox Multivariable Analysis for Overall and Cancer-Specific Survival

Characteristic	Overall survival		Cancer-specific survival	
	aHR (95% CI)	*P* value	aHR (95% CI)	*P* value
NLR				
Low	1 [Reference]	NA	1 [Reference]	NA
High	1.97 (1.26-3.09)	.003	2.33 (1.38-3.95)	.002
Gender				
Male	1 [Reference]	NA	1 [Reference]	NA
Female	0.86 (0.56-1.32)	.49	0.72 (0.41-1.26)	.25
Smoker				
Never	1 [Reference]	NA	1 [Reference]	NA
Current	2.02 (1.22-3.35)	.006	1.73 (0.90-3.30)	.1
Former	1.2 (0.78-1.85)	.41	1.22 (0.69-2.14)	.5
Age, y				
<65	1 [Reference]	NA	1 [Reference]	NA
≥65	1.89 (1.33-2.69)	<.001	1.73 (1.09-2.74)	.02
Year of radiation				
2014 or earlier	1 [Reference]	NA	1 [Reference]	NA
2015 or later	0.91 (0.63-1.32)	.62	1.09 (0.70-1.70)	.7
KPS				
<90	1 [Reference]	NA	1 [Reference]	NA
90-100	0.77 (0.53-1.11)	.16	0.46 (0.29-0.73)	<.001
Race				
White	1 [Reference]	NA	1 [Reference]	NA
Other^a^	1.41 (0.91-2.16)	.12	1.6 (0.93-2.74)	.09
Comorbidity				
0	1 [Reference]	NA	1 [Reference]	NA
1	0.63 (0.37-1.09)	.1	0.6 (0.31-1.16)	.13
2	1.02 (0.57-1.79)	.96	0.6 (0.29-1.25)	.17
3	0.43 (0.23-0.81)	.008	0.37 (0.17-0.79)	.01
>3	1.21 (0.70-2.09)	.49	0.91 (0.47-1.78)	.78
Site				
Oropharynx	1 [Reference]	NA	1 [Reference]	NA
Larynx	1.16 (0.72-1.86)	.55	1.32 (0.72-2.40)	.37
Oral cavity	1.32 (0.60-2.94)	.49	2.17 (0.84-5.62)	.11
Other	1.03 (0.66-1.62)	.9	1.39 (0.77-2.49)	.27
T staging				
1-2	1 [Reference]	NA	1 [Reference]	NA
3-4	2.40 (1.70-3.40)	<.001	3.85 (2.41-6.14)	<.001
N staging				
0	1 [Reference]	NA	1 [Reference]	NA
1	1.89 (1.02-3.50)	.04	2.35 (1.05-5.28)	.04
2	2.06 (1.26-3.36)	.004	3.46 (1.79-6.68)	<.001
3	5.43 (2.81-10.51)	<.001	8.41 (3.47-20.36)	<.001
HPV				
Negative	1 [Reference]	NA	1 [Reference]	NA
Positive	0.67 (0.41-1.11)	.12	0.71 (0.38-1.34)	.29
Chemotherapy				
Cisplatin	1 [Reference]	NA	1 [Reference]	NA
Other	1.45 (0.94-2.25)	.09	1.41 (0.79-2.50)	.24

Abbreviations: aHR, adjusted hazard ratio; HPV, human papillomavirus; KPS, Karnofsky performance status; NA, not applicable; NLR, neutrophil-lymphocyte ratio.

^a^ The other category for race and ethnicity included African American, American Indian or Alaska Native, Asian, Hispanic, and those who were unknown or declined to answer.

On logistic MVA, patients were more likely to have high NLR if they had higher T and N staging (T3-4: aOR, 4.07; 95% CI, 1.92-9.16, *P* < .001; N2: aOR, 2.97; 95% CI, 1.04-9.17; *P* = .049; N3: aOR, 11.21; 95% CI, 2.84-46.97; *P* < .001), but less likely if they had a good performance status (KPS 90-100: aOR, 0.29; 95% CI, 0.14-0.59; *P* < .001) (Table 3). A total of 331 patients (66.7%) had available HPV data. Of these, 239 patients (72.2%) had HPV-associated head and neck cancers. Median (IQR) follow-up was 43.7 (22.5-71.3) months. On Cox MVA, high NLR was not associated with OS (HPV-negative: aHR, 2.46; 95% CI, 0.96-6.31; *P* = .06; HPV-positive: aHR, 1.17; 95% CI, 0.38-3.56; *P* = .78) and CSS (HPV-negative: aHR, 2.55; 95% CI, 0.81-7.99; *P* = .11; HPV-positive: aHR, 1.45; 95% CI, 0.44-4.76; *P* = .54).

**Table 3.** Logistic Multivariable Analysis for High Neutrophil-Lymphocyte Ratio

Characteristic	aOR (95% CI)	*P* value
Gender		
Male	1 [Reference]	NA
Female	2.18 (0.95-4.91)	.06
Smoker		
Never	1 [Reference]	NA
Current	1.50 (0.50-4.64)	.47
Former	1.18 (0.46-3.26)	.73
Age, y		
<65	1 [Reference]	NA
≥65	0.46 (0.20-1.03)	.07
Year of radiation		
2014 or earlier	1 [Reference]	NA
2015 or later	0.98 (0.48-1.99)	.96
KPS		
<90	1 [Reference]	NA
90-100	0.29 (0.14-0.59)	<.001
Race		
White	1 [Reference]	NA
Other^a^	0.63 (0.20-1.68)	.38
Comorbidity		
0	1 [Reference]	NA
1	0.66 (0.23-1.97)	.45
2	1.38 (0.47-4.15)	.56
3	0.18 (0.04-0.70)	.02
>3	0.37 (0.11-1.19)	.10
Site		
Oropharynx	1 [Reference]	NA
Larynx	1.30 (0.46-3.61)	.62
Oral cavity	1.88 (0.20-11.95)	.54
Other	0.81 (0.27-2.19)	.68
T staging		
1-2	1 [Reference]	NA
3-4	4.07 (1.92-9.16)	<.001
N staging		
0	1 [Reference]	NA
1	2.16 (0.55-8.10)	.26
2	2.97 (1.04-9.17)	.049
3	11.21 (2.84-46.97)	<.001
HPV		
Negative	1 [Reference]	NA
Positive	0.45 (0.16-1.19)	.11
Chemotherapy		
Cisplatin	1 [Reference]	NA
Other	4.24 (1.74-10.36)	.001

Abbreviations: aOR, adjusted odds ratio; HPV, human papillomavirus; KPS, Karnofsky performance status; NA, not applicable.

^a^ The other category for race and ethnicity included African American, American Indian or Alaska Native, Asian, Hispanic, and those who were unknown or declined to answer.

## Discussion

To our knowledge, this is the largest study of US head and neck cancer patients who underwent definitive chemoradiation to evaluate the association between NLR and survival outcomes. Elevated NLR was an independent, adverse prognostic factor for both OS and CSS. Furthermore, it was associated with performance status and tumor staging.

The association of high NLR with worse survival in our study was consistent with a growing body of literature.^17^ Immunosuppressive neutrophils have been implicated in tumorigenesis and tumor progression,^31,32,33,34,35^ by remodeling tumor microenvironment, increasing tumor cell survival by facilitating angiogenesis, and protecting tumor cells from cytotoxic activity of lymphocytes.^36,37^ Specifically, tumor-associated neutrophils facilitate tumor growth by immunoediting,^38^ increasing proteases to facilitate tumor invasion,^39^ and activating neutrophil extracellular traps to enhance tumor adhesion and metastasis.^40^ Reduction of such tumor-associated neutrophils was shown to inhibit tumor growth, reduce immunosuppression in tumor microenvironment, and improve CD8+ cytotoxic T lymphocytes.^41,42,43^ More recently, protumorigenic vs antitumorigenic phenotypes of tumor-associated neutrophils were found to be mediated by cytokines, such as interferon beta and transforming growth factor beta,^41,44^ and the dynamic role of tumor-associated neutrophils in the context of tumor biology and microenvironment is currently evolving.^45^

Consistent with prior studies,^46,47^ we found that patients with higher disease burden were more likely to have elevated NLR. Similarly, elevated NLR was associated with worse performance status and the use of chemotherapy agents other than cisplatin in our study. Patients who are unsuitable to tolerate toxicity and morbidity from platinum-based chemotherapy may undergo other systemic therapy agents,^48^ and this association with poor performance status is consistent with elevated NLR associated with malnutrition, weight loss, and cancer cachexia.^49^ Elevated NLR remained independently associated with OS and CSS even after adjusting for these and other factors.

Among patients with available HPV data, our study found that high NLR was not associated with survival. This finding is consistent with a prior report.^50^ In contrast, other studies found that high NLR was an adverse prognostic factor for survival outcomes even in the HPV era.^18,51,52,53,54^ In addition, another study suggested HPV-associated head and neck cancers were also less likely to have high NLR,^53^ which was not observed in our study. These discrepancies may be due to the heterogeneous nature of tumor biology among HPV-associated head and neck cancers based on smoking history.^30^ Nearly 80% of patients in our study were either former or current smokers, and smoking has been shown to alter tumor gene expressions and tumor microenvironment, leading to changes in inflammation and immune-related pathways.^55,56^

### Limitations

This study has limitations, including those inherent in retrospective reviews. The neutrophils from our study were not isolated for further characterization of their phenotypes, and the heterogeneity of protumorigenic and antitumorigenic neutrophil phenotypes could not be evaluated in our study. Although several studies showed a prognostic role of dynamic changes in NLR in various cancers,^57,58,59,60^ our data on NLR after radiation therapy were missing in many patients and were not included for analysis in this study. In addition, the association between low NLR and survival would warrant further investigations, because febrile neutropenia may occur up to 15% with concurrent cisplatin.^61^ Toxicity profiles including infection and febrile neutropenia were unavailable for analysis in our study. Furthermore, most patients in our study were White individuals treated with chemoradiation. Our findings may not be generalizable to other populations with different treatment modalities and racial backgrounds.^18,19,20,24^

## Conclusions

Our study’s findings suggested that high NLR was an independent adverse prognostic factor for survival outcomes among patients with head and neck cancer undergoing chemoradiation. Patients with substantial disease burden and poor performance status were more likely to have high NLR. Further studies would be warranted to tailor treatments based on the risk stratification by NLR.

## References

[1] Hanahan D, Weinberg RA. Hallmarks of cancer: the next generation. Cell. 2011;144(5):646-674. doi:10.1016/j.cell.2011.02.013 21376230

[2] Templeton AJ, McNamara MG, Šeruga B, . Prognostic role of neutrophil-to-lymphocyte ratio in solid tumors: a systematic review and meta-analysis. J Natl Cancer Inst. 2014;106(6):dju124. doi:10.1093/jnci/dju124 24875653

[3] Dumitru CA, Lang S, Brandau S. Modulation of neutrophil granulocytes in the tumor microenvironment: mechanisms and consequences for tumor progression. Semin Cancer Biol. 2013;23(3):141-148. doi:10.1016/j.semcancer.2013.02.005 23485549

[4] Ocana A, Nieto-Jiménez C, Pandiella A, Templeton AJ. Neutrophils in cancer: prognostic role and therapeutic strategies. Mol Cancer. 2017;16(1):137. doi:10.1186/s12943-017-0707-7 28810877 PMC5558711

[5] Tazzyman S, Niaz H, Murdoch C. Neutrophil-mediated tumour angiogenesis: subversion of immune responses to promote tumour growth. Semin Cancer Biol. 2013;23(3):149-158. doi:10.1016/j.semcancer.2013.02.003 23410638

[6] Bekes EM, Schweighofer B, Kupriyanova TA, . Tumor-recruited neutrophils and neutrophil TIMP-free MMP-9 regulate coordinately the levels of tumor angiogenesis and efficiency of malignant cell intravasation. Am J Pathol. 2011;179(3):1455-1470. doi:10.1016/j.ajpath.2011.05.031 21741942 PMC3157227

[7] Cools-Lartigue J, Spicer J, McDonald B, . Neutrophil extracellular traps sequester circulating tumor cells and promote metastasis. J Clin Invest. 2013;67484. doi:10.1172/JCI67484 23863628 PMC3726160

[8] Demers M, Wagner DD. Neutrophil extracellular traps: a new link to cancer-associated thrombosis and potential implications for tumor progression. Oncoimmunology. 2013;2(2):e22946. doi:10.4161/onci.22946 23526174 PMC3601165

[9] Donskov F. Immunomonitoring and prognostic relevance of neutrophils in clinical trials. Semin Cancer Biol. 2013;23(3):200-207. doi:10.1016/j.semcancer.2013.02.001 23403174

[10] Gregory AD, Houghton AM. Tumor-associated neutrophils: new targets for cancer therapy. Cancer Res. 2011;71(7):2411-2416. doi:10.1158/0008-5472.CAN-10-2583 21427354

[11] Tazzyman S, Lewis CE, Murdoch C. Neutrophils: key mediators of tumour angiogenesis. Int J Exp Pathol. 2009;90(3):222-231. doi:10.1111/j.1365-2613.2009.00641.x 19563607 PMC2697547

[12] Cho JK, Kim MW, Choi IS, . Optimal cutoff of pretreatment neutrophil-to-lymphocyte ratio in head and neck cancer patients: a meta-analysis and validation study. BMC Cancer. 2018;18(1):969. doi:10.1186/s12885-018-4876-6 30309318 PMC6182814

[13] Kumarasamy C, Tiwary V, Sunil K, . Prognostic utility of platelet-lymphocyte ratio, neutrophil-lymphocyte ratio and monocyte-lymphocyte ratio in head and neck cancers: a detailed PRISMA compliant systematic review and meta-analysis. Cancers (Basel). 2021;13(16):4166. doi:10.3390/cancers13164166 34439320 PMC8393748

[14] Mascarella MA, Mannard E, Silva SD, Zeitouni A. Neutrophil-to-lymphocyte ratio in head and neck cancer prognosis: a systematic review and meta-analysis. Head Neck. 2018;40(5):1091-1100. doi:10.1002/hed.25075 29356179

[15] Takenaka Y, Oya R, Kitamiura T, . Prognostic role of neutrophil-to-lymphocyte ratio in head and neck cancer: A meta-analysis. Head Neck. 2018;40(3):647-655. doi:10.1002/hed.24986 29076207

[16] Tham T, Bardash Y, Herman SW, Costantino PD. Neutrophil-to-lymphocyte ratio as a prognostic indicator in head and neck cancer: a systematic review and meta-analysis. Head Neck. 2018;40(11):2546-2557. doi:10.1002/hed.25324 29761587

[17] Yang L, Huang Y, Zhou L, Dai Y, Hu G. High pretreatment neutrophil-to-lymphocyte ratio as a predictor of poor survival prognosis in head and neck squamous cell carcinoma: systematic review and meta-analysis. Head Neck. 2019;41(5):1525-1535. doi:10.1002/hed.25583 30597654 PMC6590244

[18] Gorphe P, Chekkoury Idrissi Y, Tao Y, . Anemia and neutrophil-to-lymphocyte ratio are prognostic in p16-positive oropharyngeal carcinoma treated with concurrent chemoradiation. Papillomavirus Res. 2018;5:32-37. doi:10.1016/j.pvr.2017.12.002 29253748 PMC5886959

[19] Muhaxheri G, Vucicevic Boras V, Fucic A, . Multivariate analysis of preoperative and postoperative neutrophil-to-lymphocyte ratio as an indicator of head and neck squamous cell carcinoma outcome. Int J Oral Maxillofac Surg. 2018;47(8):965-970. doi:10.1016/j.ijom.2018.02.011 29559186

[20] Azab B, Camacho-Rivera M, Taioli E. Average values and racial differences of neutrophil lymphocyte ratio among a nationally representative sample of United States subjects. PLoS One. 2014;9(11):e112361. doi:10.1371/journal.pone.0112361 25375150 PMC4223021

[21] Anantharaman D, Abedi-Ardekani B, Beachler DC, . Geographic heterogeneity in the prevalence of human papillomavirus in head and neck cancer. Int J Cancer. 2017;140(9):1968-1975. doi:10.1002/ijc.30608 28108990 PMC8969079

[22] Hashibe M, Brennan P, Benhamou S, . Alcohol drinking in never users of tobacco, cigarette smoking in never drinkers, and the risk of head and neck cancer: pooled analysis in the International Head and Neck Cancer Epidemiology Consortium. J Natl Cancer Inst. 2007;99(10):777-789. doi:10.1093/jnci/djk179 17505073

[23] Hashibe M, Brennan P, Chuang SC, . Interaction between tobacco and alcohol use and the risk of head and neck cancer: pooled analysis in the International Head and Neck Cancer Epidemiology Consortium. Cancer Epidemiol Biomarkers Prev. 2009;18(2):541-550. doi:10.1158/1055-9965.EPI-08-0347 19190158 PMC3051410

[24] Howard R, Scheiner A, Kanetsky PA, Egan KM. Sociodemographic and lifestyle factors associated with the neutrophil-to-lymphocyte ratio. Ann Epidemiol. 2019;38:11-21.e6. doi:10.1016/j.annepidem.2019.07.015 31481293 PMC8653546

[25] Fung-Kee-Fung SD, Hackett R, Hales L, Warren G, Singh AK. A prospective trial of volumetric intensity-modulated arc therapy vs conventional intensity modulated radiation therapy in advanced head and neck cancer. World J Clin Oncol. 2012;3(4):57-62. doi:10.5306/wjco.v3.i4.57 22553505 PMC3341741

[26] Contal C, O’Quigley J. An application of changepoint methods in studying the effect of age on survival in breast cancer. Comput Stat Data Anal. 1999;30(3):253-270. doi:10.1016/S0167-9473(98)00096-6

[27] Dell’Aquila E, Cremolini C, Zeppola T, . Prognostic and predictive role of neutrophil/lymphocytes ratio in metastatic colorectal cancer: a retrospective analysis of the TRIBE study by GONO. Ann Oncol. 2018;29(4):924-930. doi:10.1093/annonc/mdy004 29324972

[28] Howard R, Kanetsky PA, Egan KM. Exploring the prognostic value of the neutrophil-to-lymphocyte ratio in cancer. Sci Rep. 2019;9(1):19673. doi:10.1038/s41598-019-56218-z 31873162 PMC6928022

[29] Xie D, Marks R, Zhang M, Nomograms Predict Overall Survival for Patients with Small-Cell Lung Cancer Incorporating Pretreatment Peripheral Blood Markers. Journal of thoracic oncology: official publication of the International Association for the Study of Lung Cancer. 2015;10(8):1213-1220. 10.1097/JTO.0000000000000585 26200277

[30] Ang KK, Harris J, Wheeler R, . Human papillomavirus and survival of patients with oropharyngeal cancer. N Engl J Med. 2010;363(1):24-35. doi:10.1056/NEJMoa0912217 20530316 PMC2943767

[31] Jabłońska E, Kiluk M, Markiewicz W, Piotrowski L, Grabowska Z, Jabłoński J. TNF-alpha, IL-6 and their soluble receptor serum levels and secretion by neutrophils in cancer patients. Arch Immunol Ther Exp (Warsz). 2001;49(1):63-69. 11266093

[32] McCourt M, Wang JH, Sookhai S, Redmond HP. Proinflammatory mediators stimulate neutrophil-directed angiogenesis. Arch Surg. 1999;134(12):1325-1331. doi:10.1001/archsurg.134.12.1325 10593330

[33] McCourt M, Wang JH, Sookhai S, Redmond HP. Activated human neutrophils release hepatocyte growth factor/scatter factor. Eur J Surg Oncol. 2001;27(4):396-403. doi:10.1053/ejso.2001.1133 11417987

[34] Schaider H, Oka M, Bogenrieder T, . Differential response of primary and metastatic melanomas to neutrophils attracted by IL-8. Int J Cancer. 2003;103(3):335-343. doi:10.1002/ijc.10775 12471616

[35] Shamamian P, Schwartz JD, Pocock BJ, . Activation of progelatinase A (MMP-2) by neutrophil elastase, cathepsin G, and proteinase-3: a role for inflammatory cells in tumor invasion and angiogenesis. J Cell Physiol. 2001;189(2):197-206. doi:10.1002/jcp.10014 11598905

[36] el-Hag A, Clark RA. Immunosuppression by activated human neutrophils. dependence on the myeloperoxidase system. J Immunol. 1987;139(7):2406-2413. 2821114

[37] Petrie HT, Klassen LW, Kay HD. Inhibition of human cytotoxic T lymphocyte activity in vitro by autologous peripheral blood granulocytes. J Immunol. 1985;134(1):230-234. 3871101

[38] Dumitru CA, Moses K, Trellakis S, Lang S, Brandau S. Neutrophils and granulocytic myeloid-derived suppressor cells: immunophenotyping, cell biology and clinical relevance in human oncology. Cancer Immunol Immunother. 2012;61(8):1155-1167. doi:10.1007/s00262-012-1294-5 22692756 PMC11028504

[39] Dumitru CA, Gholaman H, Trellakis S, . Tumor-derived macrophage migration inhibitory factor modulates the biology of head and neck cancer cells via neutrophil activation. Int J Cancer. 2011;129(4):859-869. doi:10.1002/ijc.25991 21328346

[40] Park J, Wysocki RW, Amoozgar Z, . Cancer cells induce metastasis-supporting neutrophil extracellular DNA traps. Sci Transl Med. 2016;8(361):361ra138. doi:10.1126/scitranslmed.aag1711 27798263 PMC5550900

[41] Fridlender ZG, Sun J, Kim S, . Polarization of tumor-associated neutrophil phenotype by TGF-beta: “N1” versus “N2” TAN. Cancer Cell. 2009;16(3):183-194. doi:10.1016/j.ccr.2009.06.017 19732719 PMC2754404

[42] Nozawa H, Chiu C, Hanahan D. Infiltrating neutrophils mediate the initial angiogenic switch in a mouse model of multistage carcinogenesis. Proc Natl Acad Sci U S A. 2006;103(33):12493-12498. doi:10.1073/pnas.0601807103 16891410 PMC1531646

[43] Pekarek LA, Starr BA, Toledano AY, Schreiber H. Inhibition of tumor growth by elimination of granulocytes. J Exp Med. 1995;181(1):435-440. doi:10.1084/jem.181.1.435 7807024 PMC2191807

[44] Jablonska J, Leschner S, Westphal K, Lienenklaus S, Weiss S. Neutrophils responsive to endogenous IFN-beta regulate tumor angiogenesis and growth in a mouse tumor model. J Clin Invest. 2010;120(4):1151-1164. doi:10.1172/JCI37223 20237412 PMC2846036

[45] Piccard H, Muschel RJ, Opdenakker G. On the dual roles and polarized phenotypes of neutrophils in tumor development and progression. Crit Rev Oncol Hematol. 2012;82(3):296-309. doi:10.1016/j.critrevonc.2011.06.004 21798756

[46] Cho Y, Kim JW, Yoon HI, Lee CG, Keum KC, Lee IJ. The prognostic significance of neutrophil-to-lymphocyte ratio in head and neck cancer patients treated with radiotherapy. J Clin Med. 2018;7(12):E512. doi:10.3390/jcm7120512 30513928 PMC6306798

[47] Panje C, Riesterer O, Glanzmann C, Studer G. Neutrophil-lymphocyte ratio complements volumetric staging as prognostic factor in patients treated with definitive radiotherapy for oropharyngeal cancer. BMC Cancer. 2017;17(1):643. doi:10.1186/s12885-017-3590-0 28893236 PMC5594523

[48] Ahn MJ, D’Cruz A, Vermorken JB, . Clinical recommendations for defining platinum unsuitable head and neck cancer patient populations on chemoradiotherapy: a literature review. Oral Oncol. 2016;53:10-16. doi:10.1016/j.oraloncology.2015.11.019 26712252

[49] Barker T, Fulde G, Moulton B, Nadauld LD, Rhodes T. An elevated neutrophil-to-lymphocyte ratio associates with weight loss and cachexia in cancer. Sci Rep. 2020;10(1):7535. doi:10.1038/s41598-020-64282-z 32371869 PMC7200806

[50] Rosculet N, Zhou XC, Ha P, . Neutrophil-to-lymphocyte ratio: prognostic indicator for head and neck squamous cell carcinoma. Head Neck. 2017;39(4):662-667. doi:10.1002/hed.24658 28075517

[51] Kreinbrink PJ, Li J, Parajuli S, . Pre-treatment absolute lymphocyte count predicts for improved survival in human papillomavirus (HPV)-driven oropharyngeal squamous cell carcinoma. Oral Oncol. 2021;116:105245. doi:10.1016/j.oraloncology.2021.105245 33901866

[52] Ng SP, Bahig H, Jethanandani A, . Prognostic significance of pre-treatment neutrophil-to-lymphocyte ratio (NLR) in patients with oropharyngeal cancer treated with radiotherapy. Br J Cancer. 2021;124(3):628-633. doi:10.1038/s41416-020-01106-x 33051590 PMC7851392

[53] Rachidi S, Wallace K, Wrangle JM, Day TA, Alberg AJ, Li Z. Neutrophil-to-lymphocyte ratio and overall survival in all sites of head and neck squamous cell carcinoma. Head Neck. 2016;38(suppl 1):E1068-E1074. doi:10.1002/hed.24159 26040762 PMC4909151

[54] So YK, Lee G, Oh D, Byeon S, Park W, Chung MK. Prognostic role of neutrophil-to-lymphocyte ratio in patients with human papillomavirus-positive oropharyngeal cancer. Otolaryngol Head Neck Surg. 2018;159(2):303-309. doi:10.1177/0194599818764651 29557259

[55] de la Iglesia JV, Slebos RJC, Martin-Gomez L, . Effects of tobacco smoking on the tumor immune microenvironment in head and neck squamous cell carcinoma. Clin Cancer Res. 2020;26(6):1474-1485. doi:10.1158/1078-0432.CCR-19-1769 31848186 PMC7073297

[56] Irimie AI, Braicu C, Cojocneanu R, . Differential effect of smoking on gene expression in head and neck cancer patients. Int J Environ Res Public Health. 2018;15(7):E1558. doi:10.3390/ijerph15071558 30041465 PMC6069101

[57] Dan J, Tan J, Huang J, . The dynamic change of neutrophil to lymphocyte ratio is predictive of pathological complete response after neoadjuvant chemotherapy in breast cancer patients. Breast Cancer. 2020;27(5):982-988. doi:10.1007/s12282-020-01096-x 32306184

[58] Kim JY, Jung EJ, Kim JM, . Dynamic changes of neutrophil-to-lymphocyte ratio and platelet-to-lymphocyte ratio predicts breast cancer prognosis. BMC Cancer. 2020;20(1):1206. doi:10.1186/s12885-020-07700-9 33287745 PMC7720486

[59] Li Z, Zhao R, Cui Y, Zhou Y, Wu X. The dynamic change of neutrophil to lymphocyte ratio can predict clinical outcome in stage I-III colon cancer. Sci Rep. 2018;8(1):9453. doi:10.1038/s41598-018-27896-y 29930287 PMC6013456

[60] Zhao W, Wu Z, Li Y, . Pretreatment neutrophil-to-lymphocyte ratio and its dynamic changes are associated with the overall survival in advanced cancer patients undergoing palliative care. Sci Rep. 2016;6:31394. doi:10.1038/srep31394 27510632 PMC4980771

[61] Gillison ML, Trotti AM, Harris J, . Radiotherapy plus cetuximab or cisplatin in human papillomavirus-positive oropharyngeal cancer (NRG Oncology RTOG 1016): a randomised, multicentre, non-inferiority trial. Lancet. 2019;393(10166):40-50. doi:10.1016/S0140-6736(18)32779-X 30449625 PMC6541928

